# *Chlorella vulgaris* Extracts as Modulators of the Health Status and the Inflammatory Response of Gilthead Seabream Juveniles (*Sparus aurata*)

**DOI:** 10.3390/md20070407

**Published:** 2022-06-21

**Authors:** Bruno Reis, Lourenço Ramos-Pinto, Sara A. Cunha, Manuela Pintado, Joana Laranjeira da Silva, Jorge Dias, Luís Conceição, Elisabete Matos, Benjamín Costas

**Affiliations:** 1 Centro Interdisciplinar de Investigação Marinha e Ambiental (CIIMAR), Universidade do Porto, Avenida General Norton de Matos, Terminal de Cruzeiros do Porto de Leixões, 4450-208 Matosinhos, Portugal; lourenco.pinto@ciimar.up.pt; 2 SPAROS Lda., Área Empresarial de Marim, Lote C, 8700-221 Olhão, Portugal; jorgedias@sparos.pt (J.D.); luisconceicao@sparos.pt (L.C.); 3 Sorgal S.A., EN 109-Lugar da Pardala, 3880-728 São João de Ovar, Portugal; 4 Instituto de Ciências Biomédicas Abel Salazar (ICBAS-UP), Universidade do Porto, Rua de Jorge Viterbo Ferreira 228, 4050-313 Porto, Portugal; 5 CBQF—Centro de Biotecnologia e Química Fina—Laboratório Associado, Escola Superior de Biotecnologia, Universidade Católica Portuguesa Rua Diogo Botelho 1327, 4169-005 Porto, Portugal; scunha@ucp.pt (S.A.C.); mpintado@ucp.pt (M.P.); 6 Allmicroalgae, Natural Products SA, Industrial Microalgae Production, Apartado 9, 2449-909 Pataias, Portugal; joana.g.silva@allmicroalgae.com; 7 B2E Associação para a Bioeconomia Azul—Laboratório Colaborativo, Av. Liberdade, UPTEC Mar, 4450-718 Leça da Palmeira, Portugal; ematos@b2e.pt

**Keywords:** functional feeds, protein hydrolysate, innate immunity, fish robustness

## Abstract

This study aimed to evaluate the effects of short-term supplementation, with 2% *Chlorella vulgaris* (*C. vulgaris*) biomass and two 0.1% *C. vulgaris* extracts, on the health status (experiment one) and on the inflammatory response (experiment two) of gilthead seabream (*Sparus aurata*). The trial comprised four isoproteic (50% crude protein) and isolipidic (17% crude fat) diets. A fishmeal-based (FM), practical diet was used as a control (CTR), whereas three experimental diets based on CTR were further supplemented with a 2% inclusion of C. vulgaris biomass (Diet D1); 0.1% inclusion of C. vulgaris peptide-enriched extract (Diet D2) and finally a 0.1% inclusion of C. vulgaris insoluble fraction (Diet D3). Diets were randomly assigned to quadruplicate groups of 97 fish/tank (IBW: 33.4 ± 4.1 g), fed to satiation three times a day in a recirculation seawater system. In experiment one, seabream juveniles were fed for 2 weeks and sampled for tissues at 1 week and at the end of the feeding period. Afterwards, randomly selected fish from each group were subjected to an inflammatory insult (experiment two) by intraperitoneal injection of inactivated gram-negative bacteria, following 24 and 48 h fish were sampled for tissues. Blood was withdrawn for haematological procedures, whereas plasma and gut tissue were sampled for immune and oxidative stress parameters. The anterior gut was also collected for gene expression measurements. After 1 and 2 weeks of feeding, fish fed D2 showed higher circulating neutrophils than seabream fed CTR. In contrast, dietary treatments induced mild effects on the innate immune and antioxidant functions of gilthead seabream juveniles fed for 2 weeks. In the inflammatory response following the inflammatory insult, mild effects could be attributed to *C. vulgaris* supplementation either in biomass form or extract. However, the *C. vulgaris* soluble peptide-enriched extract seems to confer a protective, anti-stress effect in the gut at the molecular level, which should be further explored in future studies.

## 1. Introduction

In intensive farming facilities, fish are reared at high densities, which may increase stress and susceptibility to diseases, resulting in lower production yields. Consequently, there is an increasing pressure for disease management strategies, beyond the use of antibiotics or vaccination. In this sense, health promoting feeds designed not only to fulfil the nutrient requirements but also to strengthen the immune system are viewed as a way to reduce aquaculture dependency on chemotherapeutics and to mitigate its negative environmental effects [1,2]. Novel applications based on algal products are a fast emerging and a developing area, expected to reach 56.5 billion US$ by 2027 with a compound annual growth rate of 6% in the period from 2019 to 2027 [3]. The ability to grow in different environments and conditions as well as to produce large numbers of secondary metabolites makes microalgae a suitable raw material for different applications. These organisms are regarded as sustainable alternative sources of bioactive compounds, mostly sought out for the development of functional feeds, foods and health products [4,5,6].

*Chlorella vulgaris* is a green microalga with a wide distribution in freshwater, marine and terrestrial environments that is capable of rapid growth under autotrophic, mixotrophic and heterotrophic conditions [7]. These characteristics made *C. vulgaris* a successful candidate for large-scale cultivation and commercial production [8]. As with other microalgae species, *C. vulgaris* produces a different array of health-promoting biomolecules [9,10]. Notably, natural pigments such as lutein and astaxanthin extracted from *Chlorella* sp. show immunostimulatory and antioxidant protective effects [4,11,12]. Furthermore, these microalgae are characterised by a very high crude protein content (>50%) and a balanced amino acid (AA) profile, synthesising all essential AA in a considerable amount [4]. Already, *C. vulgaris* biomass has been successfully used in aquafeeds as a source of protein, improving growth performance, oxidative status and immune response in several fish species [13,14,15,16,17]. For instance, dietary supplementation of *Chlorella* *sp.* at 0.4 to 1.2%, stimulated the innate immunity of gibel carp (*Carassius auratus gibelio*), namely by increasing IgM, IgD, Interleukin-22 and chemokine levels [18]. Also, Zahran and Risha [16] reported that feed supplementation with powdered *C. vulgaris* protected Nile tilapia against arsenic-induced immunosuppression and oxidative stress.

Nonetheless, as with other algal biomasses, at high fishmeal replacement levels, studies start to report impaired growth performances [19,20]. Microalgae generally show thick cell walls that hinder the access of fish gut enzymes to intracellular nutrients. Hence, algae nutritional value increases if access is provided to macro and micronutrients [21,22,23]. Hydrolyses improve digestibility through the application of chemical or enzymatic methods to disrupt the cell wall and hydrolyse intact proteins [24]. The enzymatic method is sometimes advantageous because of milder processing conditions and peptide bond specificity, giving rise to digestible peptides believed to be more effective than the whole protein or the free AA [24,25]. Peptide bioactivity is influenced by molecular weight and peptide chain size [26]. In fact, low molecular weight peptides (<3 kDa) are described as having immune-stimulating or anti-inflammatory properties [26,27,28].

Several studies, have evaluated marine protein hydrolysates (MPH) as a dietary ingredient and their effects on growth performance, immune response and disease resistance in fish [26]. Results are promising, as the dietary inclusion of MPH has been shown to induce growth, antioxidant activity and fish immunity [28,29,30,31,32] as well as improve fish immune response and disease resistance to specific bacterial infections [27,33,34,35]. Moreover, regarding microalgae, different *C. vulgaris* protein hydrolysates and extracts have already been studied concerning its different bioactivities, namely, anticancer and antibacterial effects [36], as well as antioxidant and immune modulatory properties [37]. Results mentioned above suggest that *C. vulgaris* has the potential to act as a dietary supplement with nutraceutical properties and to stimulate the immune system. Therefore, the present study aimed to evaluate the effects of short-term dietary supplementation, with a 2% *C. vulgaris* biomass and a 0.1% supplementation with *C. vulgaris* soluble peptide-enriched extract, on the immune and the oxidative stress defences (health status; experiment one) and on the inflammatory response after an inflammatory insult (experiment two) of gilthead seabream (*Sparus aurata*).

## 2. Results

### 2.1. Haematology/Peripheral Leucocyte Responses

In experiment one, total WBC and RBC as well as MCH did not change significantly among different dietary treatments at both 1 and 2 weeks of feeding (Table 1). However, fish fed D2 presented a higher haemoglobin (Hb) concentration than D1 and D3 fed fish (Table 1). Differential leucocyte counts showed different modulation patterns between dietary treatments regardless of the sampling point (Table 2). For instance, the D1 fed group showed lower lymphocyte numbers at both 1 and 2 weeks, when compared to the other dietary treatments (Table 2). Whereas peripheral neutrophils increased in D2 fed fish compared to those fed CTR (Table 2). Circulating monocytes were not significantly modulated by dietary treatments at either 1 or 2 weeks of feeding.

After the inflammatory insult (experiment two), Hb increased at 24 h following inoculation with the inactivated bacteria, while MCH, total WBC and RBC remained unchanged (Table 3). Peripheral lymphocyte numbers decreased at 24 h compared to 0 h, returning to resting values at 48 h (Table 4). Circulating neutrophil levels increased at 24 h and 48 h following pathogen inoculation compared to time 0 h (Table 4). Total thrombocyte and monocyte concentrations were unaffected (Table 4).

### 2.2. Plasma Humoral Parameters

**Figure 1:**
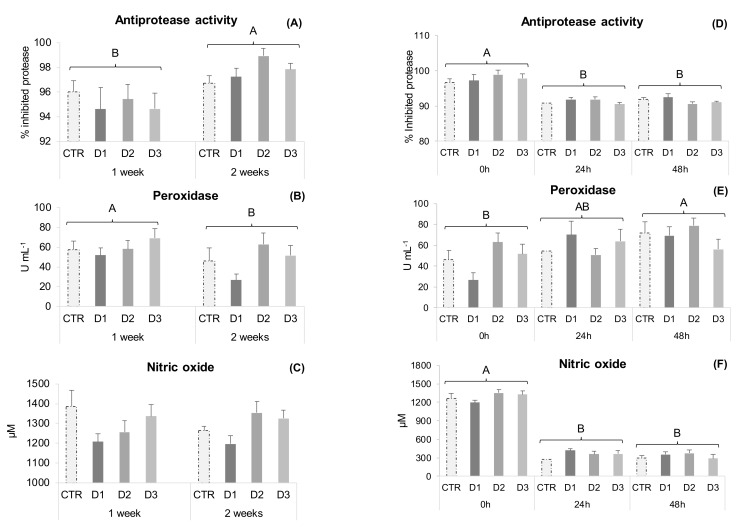
Plasma immune parameters of gilthead seabream juveniles. Experiment one: (**A**) Antiprotease activity; (**B**) Peroxidase activity; (**C**) Nitric oxide. Data are the mean ± SEM (*n* = 12). Experiment two: (**D**) Antiprotease activity; (**E**) Peroxidase activity; (**F**) Nitric oxide. Data are the mean ± SEM (*n* = 9). Different capital letters represent significant differences in time regardless diet (*p* < 0.05).

In experiment one, plasma humoral parameters (NO production, antiprotease and peroxidase activities) remained unaffected by the different dietary treatments at both sampling points (Figure 1A–C). However, antiprotease activity increased from 1 to 2 weeks of feeding, while peroxidase followed an opposite trend.

Following heat-inactivated bacteria inoculation, peroxidase activity increased after 48 h (Figure 1E), while both NO concentration and antiprotease activity decreased at 24 and 48 h (Figure 1D,F).

### 2.3. Gut Innate Immune and Oxidative Stress Biomarkers

**Figure 2:**
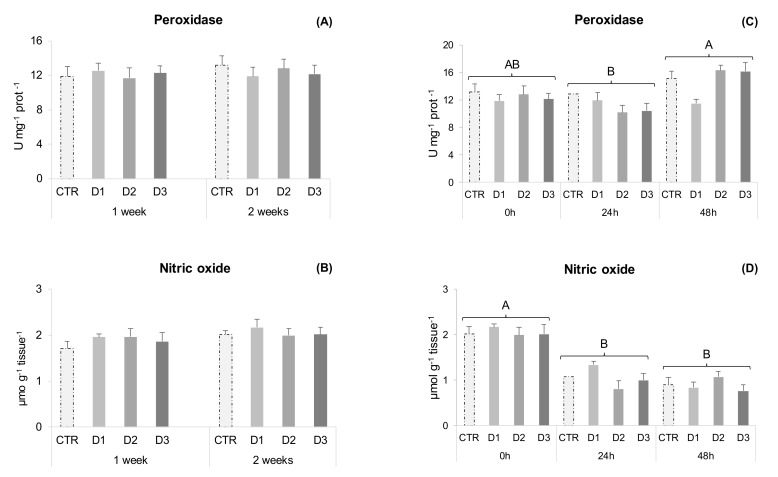
Gut immune parameters of gilthead seabream juveniles. Experiment one: (**A**) Peroxidase activity; (**B**) Nitric oxide (NO). Data are the mean ± SEM (*n* = 12). Experiment two: (**C**) Peroxidase activity; (**D**) Nitric oxide (NO). Data are the mean ± SEM (*n* = 9) Different capital letters represent significant differences in time regardless of diet (*p* < 0.05).

**Figure 3:**
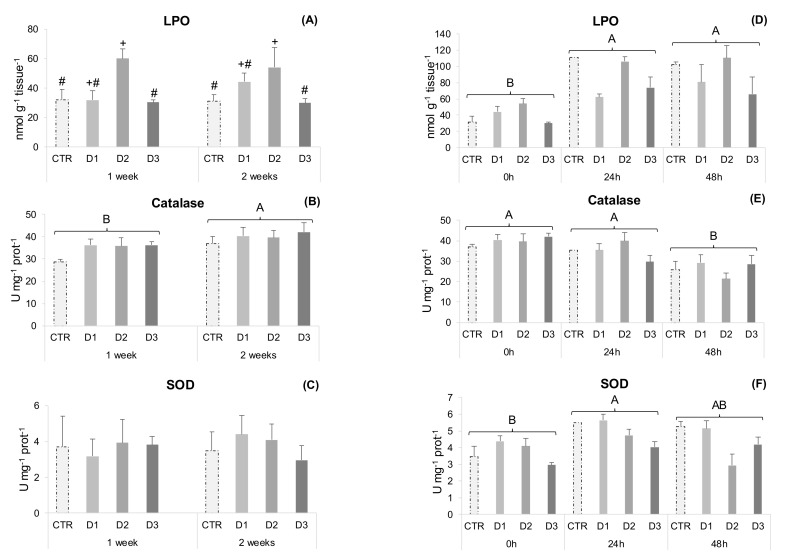
Gut oxidative stress parameters of gilthead seabream juveniles. Experiment one: (**A**) Lipid peroxidation (LPO); (**B**) Catalase activity; (**C**) Superoxide dismutase activity (SOD). Data are the mean ± SEM (*n* = 12). Experiment two: (**D**) Lipid peroxidation (LPO); (**E**) Catalase activity; (**F**) Superoxide dismutase activity (SOD). Data are the mean ± SEM (*n* = 9). Different symbols represent significant differences between diets regardless of time (*p* < 0.05). Different capital letters represent significant differences in time regardless of diet (*p* < 0.05).

Peroxidase, NO production and SOD activity remained unchanged during the health status experiment in gut samples (Figure 2A–C). Nonetheless, D2 fed fish showed increased gut lipid peroxidation compared to D3 and CTR (Figure 3A), and catalase activity increased from 1 to 2 weeks of feeding.

In experiment two, all measured parameters changed over time. Peroxidase activity increased from 24 to 48 h and NO production decreased after 24 and 48 h (Figure 2C,D). Antioxidant defences, such as catalase activity decreased 48 h after inoculation (Figure 3E), while lipid peroxidation increased at 24 and 48 h (Figure 3D). Superoxide dismutase activity increased at 24 h post-stimulus and D1 fed fish had higher activity than D3, irrespective of the sampling point (Figure 3F).

### 2.4. Gut Gene Expression Analysis

To evaluate the expression of gut health, immunity and oxidative stress related genes (Table 5 and Table 6), total RNA was isolated from fish anterior intestine. In experiment one, target genes transcriptomic analysis was not able to ascertain differences attributable to the dietary treatments, which could be related to the high intraspecific variability for some target genes (Table 5). However, cd8α, hsp70 and muc2 genes expression increased from 1 to 2 weeks.

Following the inflammatory insult, changes attributed to dietary treatments were also not found in the majority of analysed genes, except for hsp70, which was down-regulated at 24 h in D2 fed fish (Table 6). Furthermore, tlr1 gene expression was up-regulated and gpx was down-regulated at 24 h in all dietary treatments.

## 3. Discussion

A main feature of *C. vulgaris* is its protein content and its balanced AA profile, making it a potential source of bioactive peptides. However, the presence of rigid cell walls limits the fish’s ability to access and to utilise the different nutrients inside microalgae cells. In the present study, cell wall disruption was obtained through a combination of chemical and enzymatic processes and the protein fraction was hydrolysed using a serine protease. Protein hydrolysates seem more effective than either intact protein or free AA in different applications for nutrition [25,38]. The current study was devised using two different approaches. First, there was a 2-week feeding trial to evaluate the health status of the fish, aiming to develop future prophylactic strategies (experiment one). After 2 weeks of feeding, fish were subjected to an inflammatory insult to evaluate the inflammatory response (experiment two) and to better discriminate any immunomodulatory effect from the different dietary treatments.

The overall haematological profile from the health status experiment showed some changes, mainly exerted by *C. vulgaris* biomass and peptide-enriched extract supplemented diets (D1 and D2 diets). Fish fed diet D1 showed lower lymphocyte numbers (Table 2). Accordingly, in a previous experiment with poultry, where different preparations of *C. vulgaris* were used, animals fed a supplemented diet with 1% chlorella powder showed decreased lymphocyte numbers [39]. Nonetheless, fish fed D2 diet not only had comparable lymphocyte numbers to CTR, but also showed a higher neutrophil concentration (Table 2). These higher circulating myeloid cell numbers in the D2 group might be of relevance during early responses to infection. Bøgwald et al. [40] have shown that medium-size peptides (500–3000 Da) from cod muscle protein hydrolysate, stimulated in vivo respiratory burst activity in Atlantic salmon (*Salmo salar*) head-kidney leucocytes. In the present study, the peptide-enriched extract protein/peptide profile (Figure S1) is mainly composed of small to medium size particles (<1200 Da) [41]. Size and molecular weight (MW) seem to be particularly important for peptide immunomodulatory activities, with small- to medium-sized particles showing the highest activity [26,28,40,42]. However, an increased leucocyte response in fish fed the D2 diet did not translate into an improved plasma humoral parameters response (NO concentration, antiprotease and peroxidase activities) at 1 or 2 weeks (Figure 1A–C), although those values tended to increase in seabream fed D2 and D3. Accordingly, former studies conducted on Coho salmon (*Oncorhynchus kisutch*) and turbot (*Scophthalmus maximus*) did not show any significant impacts on several innate immune defence mechanisms, when fish were fed MPH supplemented diets [43,44]. Nonetheless, beneficial effects have been reported in different fish species [26]. Khosravi et al. [33] supplemented red seabream (*Pagrus major*) and olive flounder (*Paralichthys olivaceus*) feeds with 2% krill and tilapia protein hydrolysates and supplementation improved lysozyme activity and respiratory burst in both species. Protein hydrolysates were mainly composed of small- (<500 Da) to medium-sized peptides (500–5000 Da). Furthermore, diet D2 shows a higher Hb concentration than D1 and D3 fed fish. The extraction method employed in a *C. vulgaris* biomass to obtain the soluble extract (diet D2) might increase iron availability, since most of the intracellular iron is associated with soluble proteins and iron is an essential element for Hb production [45].

In the present study, when fish were subjected to an inflammatory insult (experiment two), an immune response after the stimulus was observed through the time-dependent response pattern of peripheral leucocytes, plasma and gut immune parameters. Peripheral cell dynamics were significantly changed at 24 h post-stimulus, translating into a sharp increase in circulating neutrophils and a significant decrease in lymphocytes (Table 4), indicating that cells were differentiating and being recruited to the site of inflammation. Also, Hb concentration increased (Table 3) in line with a higher metabolic expenditure due to the inflammatory response, and peroxidase activity showed a clear augmentation following inflammation (Figure 1E). Even though circulating neutrophil numbers tended to increase in D1, 2 and 3 dietary treatments at 48 h following inflammation (Table 4), it was not possible to ascertain a clear Chlorella whole-biomass or extracts effect, a fact that could be related to high intraspecific variability in response to the stimulus and that reinforces the need for further studies to unravel the potential of these extracts.

Hydrogen peroxide and oxygen radicals are physiologically generated within cellular compartments and their build-up leads to tissue oxidative stress and damage [46]. Free radical effects are controlled endogenously by antioxidant enzymes and non-enzymatic antioxidants and also by exogenous dietary antioxidants that prevent oxidative damage. Chlorella sp. contain several phytochemicals, namely carotenoids, chlorophyll, flavonoids and polyphenols, which exhibit antioxidant activities [47,48]. Earlier studies showed a significant increase in serum SOD activity in gibel carp fed diets containing 0.8–2.0% dry Chlorella powder [20]. Rahimnejad et al. [14] reported increased plasma CAT activity and total antioxidant capacity (TAC) in olive flounder fed diets with 5% and 10% defatted *C. vulgaris* meal. As with other microalgae species, the antioxidant potential of *C. vulgaris* has been mainly assessed on serum and liver, though information is still scarce at the intestinal level. The intestinal epithelium, a highly selective barrier between the animal and the external environment, is constantly exposed to dietary and environmental oxidants. Consequently, it is more prone to oxidative stress and damage, which can impact gut functionality and health [49,50]. The dietary effects of microalgae biomass inclusion have been previously assessed on the intestine of gilthead seabream. Fish were fed diets supplemented with 0.5, 0.75 and 1.5% *Nannochloropsis gaditana* biomass and no signs of nutritional modulation were found for intestinal SOD and CAT transcription [51]. In the present study, D2 fed fish showed higher gut LPO than CTR and D3 at the end of experiment one (Figure 3A), which could be related to the extraction method employed, since most of the pigments present in the *C. vulgaris* biomass are not present in the peptide-enriched extract, diminishing the availability of exogenous dietary antioxidants. As pigments are mostly hydrophobic, they are extracted alongside the lipid fraction present in the insoluble extract (Diet D3). Regarding the activities of key enzymes involved in intestinal redox homeostasis (CAT and SOD), these remained unchanged among experimental groups. Castro et al. [17] replaced 100% FM by *C. vulgaris* biomass in plant protein rich diets for seabass (*Dicentrarchus labrax*) and found no differences in intestinal LPO, tGSH and GSH levels between dietary treatments. However, they reported lower SOD activity and higher GSSG levels in microalgae-enriched diets, suggesting an increased risk for oxidative stress when fish are subjected to pro-oxidative conditions. Such conditions might arise during an inflammatory insult. However, in experiment two of the present study, lipid peroxidation increased at 24 and 48 h (Figure 3D) post-stimulus but to the same extent for all the dietary treatments. It could be hypothesised that fish fed the D2 diet were able to cope with acute inflammation in a similar manner as the other experimental groups, despite their higher intestinal oxidative state. In other studies, *C. vulgaris* powdered biomass has been found to counteract the pro-oxidative effects of arsenic induced toxicity in both the gills and the liver of tilapia [16]. Furthermore, Grammes et al. [51] reported that substituting FM by *C. vulgaris* in aquafeeds containing 20% soybean meal (SBM) is an effective strategy to counteract soybean meal-induced enteropathy (SBMIE) in Atlantic salmon. Likely, this was by maintaining the integrity of the intestinal epithelial barrier and therefore preventing innate immune response activation and ROS generation [52,53].

In the present study, anterior gut transcriptional changes were also evaluated to determine the effect of dietary treatments on the expression patterns of different structural (muc2 and muc13), antioxidant (hsp70; gpx and sod(mn)) and immune related genes (il1β; il34; tlr1; cd8α; igm and hepc). The transcriptomic approach employed was not able to ascertain a clear dietary modulation, at least for the great majority of genes under evaluation in both experiments one and two. However, after the inflammatory insult, the hsp70 gene was down regulated in the D2 fed group after 24 h compared to those fed CTRL (Table 6). Heat shock protein 70 (HSP70) maintains cell integrity and function, and it promotes cell survival under stressful conditions [54]. Leduc et al. [28] reported that genes involved in cellular damage response and repair were also under-expressed in seabass fed a mix of tilapia (TH) and shrimp (SH) protein hydrolysates (5% dry matter diet), mainly composed of low molecular weight peptides. In the same study, fish that were fed the SH alone showed up-regulation of intestinal immune-related genes. Although composed of small-sized peptides, TH did not show the same pattern of stimulation, following what was observed in the current work. According to the authors, the immune-stimulatory effect of the SH was due to low molecular weight peptides, but also to its origin and its degree of hydrolysis [28]. Bioactive peptides are inactive when they are part of the native protein sequence; and, after hydrolysis, bioactivity can be gained depending on specific AA sequences and the size of the newly formed peptides [25]. Nevertheless, in the present study, the observed down-regulation of hsp70 gene expression in the gut of seabream fed D2 suggests a certain degree of anti-stress and/or antioxidant properties from the *C. vulgaris* peptide-enriched extract, in line with that hypothesized above.

In summary, the *C. vulgaris* peptide-enriched extract tested in the present study seems to confer a dual modulatory effect at both peripheral (blood) and local (gut) levels. In particular, it drives the proliferation of circulating neutrophils in resting seabream, which could be of assistance to fight against opportunistic pathogens. Following an inflammatory insult, this peptide-enriched extract may protect the gut against stress, and it should be considered for further studies.

## 4. Materials and Methods

### 4.1. C. vulgaris Hydrolysates Production

*C. vulgaris* was supplied, as powder, by AllMicroalgae—Natural Products, SA (Pataias, Portugal). The *C. vulgaris* hydrolysates were produced by an acid pre-treatment followed by an enzymatic hydrolysis, using a previously optimised method [41]. Briefly, C. vulgaris (Table 7) was mixed with an acetic acid solution (2% in deionised water) in a ratio of microalgae:water of 1:3 (*w*/*v*). The mixture was incubated for 1 h at 50 °C and 125 rpm in an orbital shaker (ThermoFisher Scientific, Waltham, MA, USA, MaxQ™ 6000). Then, deionised water was added until microalgae:water ratio reached 1:10 and the pH was adjusted to 7.5. For the enzymatic hydrolysis, first, 5% cellulase was added and the mixture was incubated for 2 h at 50 °C and 125 rpm. Secondly, 3.9% subtilisin was added and the mixture was incubated for 2 h at 40 °C at 125 rpm. During the enzymatic hydrolysis, pH was constantly verified and adjusted to 7.5, mainly in the subtilisin hydrolysis step. To stop the hydrolysis reaction, the mixture was incubated at 90 °C for 10 min to inactivate the enzymes. The resulting solution was centrifuged at 5000× *g* for 20 min, and both the water-soluble peptide-enriched supernatant (Table 8) and the pellet were collected and freeze-dried for further analysis.

### 4.2. Diet Composition

The trial comprised 4 isoproteic (50% protein in dry matter (DM)), isolipidic (17% fat in DM) and isoenergetic (23 kJ/g) dietary treatments. A fishmeal-based (FM), practical diet was used as a control (CTR), whereas three experimental diets based on CTR were further supplemented with a 2% inclusion of *C. vulgaris* powdered biomass (Diet D1); 0.1% inclusion of *C. vulgaris* peptide-enriched extract (Diet D2) and finally 0.1% inclusion of *C. vulgaris* insoluble residue (Diet D3) (Table 9). Diets were manufactured by SPAROS (Olhão, Portugal). All powder ingredients were initially mixed and ground (<200 micron) in a micropulverizer hammer mill (SH1, Hosokawa-Alpine, Germany). Subsequently, the oils were added to the powder mixtures, which were humidified with 25% water and agglomerated by a low-shear and a low-temperature extrusion process (ITALPLAST, Parma, Italy). The resulting pellets of 2.0 mm were dried in a convection oven for 4 h at 55 °C (OP 750-UF, LTE Scientifics, Oldham, UK). Diets were packed in sealed plastic buckets and shipped to the research site (CIIMAR, Matosinhos, Portugal), where they were stored in a temperature-controlled room.

### 4.3. Bacterial Growth and Inoculum Preparation

*Photobacterium damselae* subsp. piscicida (Phdp), strain PP3, was used for the inflammatory insult. Bacteria were routinely cultured at 22 °C in tryptic soy broth (TSB) or tryptic soy agar (TSA) (both from BD Difco™, Franklin Lakes, NJ, USA) supplemented with NaCl to a final concentration of 1% (*w*/*v*) (TSB-1 and TSA-1, respectively) and stored at −80 °C in TSB-1 supplemented with 15% (*v*/*v*) glycerol. To prepare the inoculum for injection into the fish peritoneal cavities, stocked bacteria were cultured for 48 h at 22 °C on TSA-1. Afterwards, exponentially growing bacteria were collected and resuspended in sterile HBSS and adjusted against its growth curve to 1 × 10^7^ colony forming units (cfu) mL^−1^. Plating serial dilutions of the suspensions onto TSA-1 plates and counting the number of cfu following incubation at 22 °C confirmed bacterial concentration of the inocula. Bacteria were then killed by heat at 70 °C for 10 min. Loss of bacterial viability following heat exposure was confirmed by plating resulting cultures on TSA-1 plates and failing to see any bacterial growth.

### 4.4. Fish Rearing Conditions and Feeding Scheme

The experiment was carried out in compliance with the Guidelines of the European Union Council (Directive 2010/63/EU) and Portuguese legislation for the use of laboratory animals at CIIMAR aquaculture and animal experimentation facilities in Matosinhos, Portugal. The protocol was approved by the CIIMAR Animal Welfare Committee in 29/04/2020 with the reference 0421/000/000/2020 from Direção Geral de Alimentação e Veterinária (DGAV). Seawater flow was kept at 4 L min^−1^ (mean temperature 22.4 ± 1 °C; mean salinity 35.2 ± 0.7 ‰) in a recirculation system with aeration (mean dissolved oxygen above 6 mg L^−1^). Water quality parameters were monitored daily and adjusted when necessary. Mortality was monitored daily. Diets were randomly assigned to triplicate groups of 97 fish/tank (IBW: 33.4 ± 4.1 g) that were fed to satiation three times a day for 2 weeks starting at a 1.5% biomass.

### 4.5. Experimental Procedures

To examine the influence that *C. vulgaris* biomass and protein-rich extract supplementation may have on the health status (trial 1) and the inflammatory response against bacteria (inactivated Phdp i.p. injection; trial 2), samples of blood and gut were collected at 1 and 2 weeks (Trial 1) and after 2 weeks of feeding at 0 h, 24 h and 48 h post-injection (Trial 2).

#### 4.5.1. Health Status (Experiment One)

After 1 and 2 weeks, 12 fish/treatment were weighed and sampled for tissues (blood, head-kidney, liver and gut), after being sacrificed with a 2-phenoxyethanol lethal dose (0.5 mL L^−1^) [55]. Blood was collected from the caudal vein using heparinised syringes and centrifuged at 10,000× *g* for 10 min at 4 °C to obtain plasma samples. Plasma and tissue samples were immediately frozen in liquid nitrogen and stored at −80 °C until further analysis.

#### 4.5.2. Inflammatory Response (Experiment Two)

At 2 weeks, 24 fish/treatment were subjected to an inflammatory insult by intraperitoneal (i.p.) injection of heat-inactivated Phdp (see Section 2.2) and immediately transferred to a similar recirculation system in triplicates. After 24 and 48 h post-injection (time-course), 9 fish/treatment were sampled as described above.

### 4.6. Haematological Procedures

The haematological profile consisted of total white (WBC) and red (RBC) blood cells counts. To determine WBC and RBC concentration, whole blood was diluted 1/20 and 1/200, respectively, in HBSS with heparin (30 U mL^−1^) and cell counts were done in a Neubauer chamber. Blood smears were prepared from peripheral blood, air-dried and stained with Wright’s stain (Haemacolor; Merck, Darmstadt, Germany), after fixation for 1 min with formol–ethanol (10% formaldehyde in ethanol). Neutrophils were labelled by detecting peroxidase activity revealed by Antonow’s technique described in Afonso et al. [56]. The slides were examined under oil immersion (1000×), and at least 200 leucocytes were counted and classified as thrombocytes, lymphocytes, monocytes and neutrophils. The relative percentage and absolute value (×10^4^ mL^−1^) of each cell type was calculated.

### 4.7. Innate Humoral Parameters

#### 4.7.1. Antiprotease Activity

The antiprotease activity was determined as described by Ellis et al. [57], with some modifications. Briefly, 10 µL of plasma were incubated with the same volume of trypsin solution (5 mg mL^−1^ in NaHCO_3_, 5 mg mL^−1^, pH 8.3) for 10 min at 22 °C. After incubation, 100 µL of phosphate buffer (NaH_2_PO_4_, 13.9 mg mL^−1^, pH 7.0) and 125 μL of azocasein solution (20 mg mL^−1^ in NaHCO_3_, 5 mg mL^−1^, pH 8.3) were added and incubated for 1 h at 22 °C. Finally, 250 μL of trichloroacetic acid were added to the reaction mixture and incubated for 30 min at 22 °C. The mixture was centrifuged at 10,000× *g* for 5 min at room temperature. Afterwards, 100 μL of the supernatant was transferred to a 96 well-plate and mixed with 100 μL of NaOH (40 mg mL^−1^). The OD was read at 450 nm in a Synergy HT microplate reader (Biotek, Winooski, VT, USA). Phosphate buffer instead of plasma and trypsin served as blank, whereas the reference sample was phosphate buffer instead of plasma. The sample inhibition percentage of trypsin activity was calculated as follows: 100 – ((sample absorbance/Reference absorbance) × 100). All analyses were conducted in duplicates.

#### 4.7.2. Peroxidase Activity

Total peroxidase activity in plasma and intestine was measured, following the procedure described by Quade and Roth [58]. Briefly, 10 μL of plasma and 5 μL of intestine homogenate were diluted with 140 and 145 μL, respectively, of HBSS without Ca^2+^ and Mg^2+^ in 96-well plates. Then, 50 μL of 20 mM 3,3′,5,5′-tetramethylbenzidine hydrochloride (TMB; Sigma-Aldrich^®^, Merck, Darmstadt, Germany) and 50 μL of 5 mM H_2_O_2_ were added to the wells. The reaction was stopped after 2 min by adding 50 μL of H_2_SO_4_ (2 M) and the optical density (OD) was read at 450 nm in a Synergy HT microplate reader (Biotek, Winooski, VT, USA). Wells without plasma or mucus were used as blanks. The peroxidase activity (U mL^−1^ tissue) was determined, defining that one unit of peroxidase produces an absorbance change of 1 OD.

#### 4.7.3. Nitric Oxide (NO) Production

NO production was measured in plasma (1:10 sample dilution) and intestine (1:5 sample dilution) samples. Total nitrite and nitrate concentrations in the sample were assessed using the Nitrite/Nitrate colorimetric method kit (Roche, Basel, Switzerland) adapted to microplates. Nitrite concentration was calculated by comparison with a sodium nitrite standard curve. Since nitrite and nitrate are endogenously produced as oxidative metabolites of the NO molecule, these compounds are considered as indicative of NO production.

### 4.8. Analysis of Oxidative Stress Biomarkers

Intestine samples were homogenised (1:10) in phosphate buffer 0.1 M (pH 7.4), using Precellys evolution tissue lyser homogenizer (Bertin Instruments, Montigny-le-Bretonneux, France).

#### 4.8.1. Lipid Peroxidation (LPO)

One aliquot of tissue homogenate was used to determine the extent of endogenous LPO by measuring thiobarbituric acid-reactive species (TBARS) as suggested by Bird and Draper [59]. To prevent artifactual lipid peroxidation, butylhydroxytoluene (BHT 0.2 mM) was added to the aliquot. Briefly, 1 mL of 100% trichloroacetic acid and 1 mL of 0.73% thiobarbituric acid solution (in Tris–HCl 60 mM pH 7.4 with DTPA 0.1 mM) were added to 0.2 mL of intestine homogenate. After incubation at 100 °C for 60 min, the solution was centrifuged at 12,000× *g* for 5 min and LPO levels were determined at 535 nm.

#### 4.8.2. Total Protein Quantification

The remaining tissue homogenate was centrifuged for 20 min at 12,000 rpm (4 °C) to obtain the post-mitochondrial supernatant fraction (PMS). Total proteins in homogenates were measured by using Pierce™ BCA Protein Assay Kit, as described by the manufacturer (ThermoFisher Scientific, Waltham, MA, USA).

#### 4.8.3. Catalase (CAT)

CAT activity was determined in PMS by measuring substrate (H_2_O_2_) consumption at 240 nm according to Claiborne [60] adapted to microplate. Briefly, in a microplate well, 0.140 mL of phosphate buffer (0.05 M pH 7.0) and a 0.150 mL H_2_O_2_ solution (30 mM in phosphate buffer 0.05 M pH 7.0) were added to 0.01 mL of intestine PMS (0.7 mg mL^−1^ total protein). Enzymatic activity was determined in a microplate reader (BioTek Synergy HT, Winooski, VT, USA), reading the optical density at 240 nm for 2 min every 15 s interval.

#### 4.8.4. Superoxide Dismutase (SOD)

SOD activity was measured according to Flohé and Otting [61], adapted to microplate by Lima, et al. [62]. Briefly, in a microplate well, 0.2 mL of the reaction solution [1 part xanthine solution 0.7 mM (in NaOH 1 mM) and 10 parts cytochrome c solution 0.03 mM (in phosphate buffer 50 mM pH 7.8 with 1 mM Na-EDTA)] was added to 0.05 mL of intestine PMS (0.25 mg mL^−1^ total protein). Optical density was measured at 550 nm in a microplate reader (BioTek Synergy HT, Winooski, VT, USA) every 20 sec interval for 3 min at 25 °C.

### 4.9. Gene Expression

RNA isolation from target tissue (anterior gut) and cDNA synthesis was conducted with NZY Total RNA Isolation kit and NZY first-strand cDNA synthesis kit (NZYTech, Lisbon, Portugal), following manufacturer’s specifications. Real-time quantitative PCR was carried out on a CFX384 Touch Real-Time PCR system (Bio-Rad Laboratories, Hercules, CA, USA). Genes comprised in the assay were selected for their involvement in gut integrity, health and immunity (Table 10). Specific primer pair sequences are listed in Table S1. Controls of general PCR performance were included on each array. Briefly, RT reactions were diluted to obtain the equivalent concentration of 20 ng of total input RNA which were used in a 10 μL volume for each PCR reaction. PCR wells contained a 2× SYBR Green Master Mix (Bio-Rad Laboratories, Hercules, CA, USA) and specific primers were used to obtain amplicons 50–250 bp in length. The program used for PCR amplification included an initial denaturation step at 95 °C for 10 min, followed by 40 cycles of 95 °C denaturation for 15 s, with primer annealing and extension temperature (Table S1) for 1 min. The efficiency of PCR reactions was always higher than 90%, and negative controls without sample templates were routinely performed for each primer set. The specificity of reactions was verified by analysis of melting curves (ramping rates of 0.5 °C/10 s over a temperature range of 55–95 °C). Fluorescence data acquired during the PCR extension phase were normalised using the Pfaffl [63] method. The geometric mean of two carefully selected housekeeping genes (elongation factor 1-α (ef1α) and ribosomal protein S18 (rps18)) was used as the normalisation factor to normalise the expression of target genes. For comparing the mRNA expression level of each gene in a given dietary treatment, all data values were in reference to the expression level of CTR fish.

### 4.10. Data Analysis

All results are expressed as mean ± standard error (mean ± SE). Residuals were tested for normality (Shapiro–Wilk’s test) and homogeneity of variance (Levene’s test). When residuals did not meet the assumptions, data was transformed by a Log10 or square root transformation. For gene expression data, a log2 transformation was applied to all expression values. Two-way ANOVAs were performed in data arising from both trials one and two, with “dietary treatment and time” as the fixed effects. Analysis of variance was followed by Tukey post-hoc tests. All statistical analyses were performed using the computer package SPSS 26 for WINDOWS. The level of significance used was *p* ≤ 0.05 for all statistical tests.

## Figures and Tables

**Table 1:** Haemoglobin, mean corpuscular haemoglobin (MCH), red blood cells (RBC) and white blood cells (WBC) in gilthead seabream juveniles after 1 and 2 weeks of feeding (experiment one). Data are the mean ± SEM (*n* = 12).

Haematology	1 Week				2 Weeks			
Diets	CTR	D1	D2	D3	CTR	D1	D2	D3
Haemoglobin (g·dL^−1^)	0.69 ± 0.05	0.65 ± 0.04	0.81 ± 0.04	0.61 ± 0.03	0.68 ± 0.06	0.65 ± 0.02	0.74 ± 0.05	0.67 ± 0.05
MCH (pg·cell^−1^)	2.24 ± 0.31	1.89 ± 0.16	2.29 ± 0.23	1.87 ± 0.16	3.31 ± 0.39	3.12 ± 0.19	3.61 ± 0.19	3.24 ± 0.18
WBC (10^4^·μL^−1^)	1.85 ± 0.05	1.84 ± 0.12	1.96 ± 0.06	1.86 ± 0.05	3.96 ± 0.18	4.19 ± 0.21	3.73 ± 0.23	3.89 ± 0.19
RBC (10^6^·μL^−1^)	3.25 ± 0.26	3.46 ± 0.21	3.79 ± 0.39	3.64 ± 0.20	1.93 ± 0.09	2.13 ± 0.16	2.06 ± 0.12	1.91 ± 0.11
2-Way ANOVA								
	Time		Diet				Diet × Time	
	1 week	2 weeks	CTR	D1	D2	D3		
Haemoglobin (g·dL^−1^)	-	-	AB	B	A	B	ns	
MCH (pg·cell^−1^)	A	B	-	-	-	-	ns	
WBC (10^4^·μL^−1^)	B	A	-	-	-	-	ns	
RBC (10^6^·μL^−1^)	A	B	-	-	-	-	ns	

Different capital letters represent significant differences in time regardless of diet and between diets regardless of time (*p* < 0.05), ns (not significant).

**Table 2:** Absolute values of peripheral blood leucocytes (thrombocytes, Lymphocytes, monocytes and neutrophils) in gilthead seabream juveniles after 1 and 2 weeks of feeding (experiment one). Data are the mean ± SEM (*n* = 12).

Peripheral Leucocytes	1 Week				2 Weeks			
Diets	CTR	D1	D2	D3	CTR	D1	D2	D3
Thrombocytes (10^4^·μL^−1^)	1.17 ± 0.08	1.30 ± 0.07	1.29 ± 0.12	1.26 ± 0.05	2.78 ± 0.18	3.24 ± 0.17	2.58 ± 0.17	2.66 ± 0.16
Lymphocytes (10^4^·μL^−1^)	0.57 ± 0.07	0.37 ± 0.04	0.62 ± 0.05	0.56 ± 0.03	1.15 ± 0.12	0.84 ± 0.07	1.05 ± 0.15	1.17 ± 0.08
Monocytes (10^4^·μL^−1^)	0.01 ± 0.00	0.02 ± 0.00	0.02 ± 0.01	0.02 ± 0.01	0.02 ± 0.01	0.03 ± 0.01	0.02 ± 0.00	0.02 ± 0.01
Neutrophils (10^4^·μL^−1^)	0.01 ± 0.00	0.01 ± 0.01	0.04 ± 0.01	0.02 ± 0.01	0.02 ± 0.01	0.06 ± 0.02	0.06 ± 0.01	0.06 ± 0.01
2-Way ANOVA								
	Time		Diet				Diet × Time	
	1 week	2 weeks	CTR	D1	D2	D3		
Thrombocytes (10^4^·μL^−1^)	B	A	-	-	-	-	ns	
Lymphocytes (10^4^·μL^−1^)	B	A	A	B	A	A	ns	
Monocytes (10^4^·μL^−1^)	-	-	-	-	-	-	ns	
Neutrophils (10^4^·μL^−1^)	B	A	B	AB	A	AB	ns	

Different capital letters represent significant differences in time regardless of diet and between diets regardless of time (*p* < 0.05), ns (not significant).

**Table 3:** Haemoglobin, mean corpuscular haemoglobin (MCH), red blood cells (RBC) and white blood cells (WBC) in gilthead seabream juveniles following an inflammatory insult after 2 weeks of feeding (experiment two). Data are the mean ± SEM (*n* = 9).

Haematology	0 h				24 h				48 h			
Diets	CTR	D1	D2	D3	CTR	D1	D2	D3	CTR	D1	D2	D3
Haemoglobin (g·dL^−1^)	0.68 ± 0.06	0.65 ± 0.02	0.74 ± 0.05	0.67 ± 0.05	0.87 ± 0.10	0.79 ± 0.10	0.96 ± 0.08	0.78 ± 0.09	0.57 ± 0.05	0.83 ± 0.08	0.73 ± 0.05	0.69 ± 0.03
MCH (pg·cell^−1^)	3.31 ± 0.39	3.12 ± 0.19	3.61 ± 0.19	3.24 ± 0.18	3.60 ± 0.28	3.82 ± 0.33	3.98 ± 0.33	3.79 ± 0.34	3.09 ± 0.28	3.93 ± 0.33	3.43 ± 0.16	3.54 ± 0.13
WBC (10^4^·μL^−1^)	3.96 ± 0.18	4.19 ± 0.21	3.73 ± 0.23	3.89 ± 0.19	4.04 ± 0.28	4.23 ± 0.28	4.09 ± 0.42	4.11 ± 0.32	4.56 ± 0.29	4.69 ± 0.26	4.19 ± 0.35	4.42 ± 0.35
RBC (10^6^·μL^−1^)	1.93 ± 0.09	2.13 ± 0.16	2.06 ± 0.12	1.91 ± 0.11	2.20 ± 0.14	2.11 ± 0.12	2.34 ± 0.13	2.05 ± 0.09	1.86 ± 0.11	2.23 ± 0.10	2.13 ± 0.10	1.97 ± 0.09
2-Way ANOVA												
	Time				Diet				Diet × Time			
	0 h	24 h	48 h		CTR	D1	D2	D3				
Haemoglobin	B	A	B		-	-	-	-	ns			
MCH	-	-	-		-	-	-	-	ns			
WBC	-	-	-		-	-	-	-	ns			
RBC	-	-	-		-	-	-	-	ns			

Different capital letters represent significant differences in time regardless of diet (*p* < 0.05), ns (not significant).

**Table 4:** Absolute values of peripheral blood leucocytes (thrombocytes, Lymphocytes, monocytes and neutrophils) in gilthead seabream juveniles following an inflammatory insult after 2 weeks of feeding (experiment two). Data are the mean ± SEM (*n* = 9).

Peripheral Leucocytes	0 h				24 h				48 h			
Diets	CTR	D1	D2	D3	CTR	D1	D2	D3	CTR	D1	D2	D3
Thrombocytes (10^4^·μL^−1^)	2.78 ± 0.18	3.24 ± 0.17	2.58 ± 0.17	2.66 ± 0.16	3.06 ± 0.16	3.05 ± 0.24	3.33 ± 0.29	3.26 ± 0.28	3.14 ± 0.33	3.10 ± 0.16	2.88 ± 0.19	2.93 ± 0.20
Lymphocytes (10^4^·μL^−1^)	1.15 ± 0.12	0.84 ± 0.07	1.05 ± 0.15	1.17 ± 0.08	0.75 ± 0.13	0.88 ± 0.13	0.76 ± 0.14	0.60 ± 0.10	1.09 ± 0.12	1.00 ± 0.12	0.93 ± 0.15	0.87 ± 0.07
Monocytes (10^4^·μL^−1^)	0.02 ± 0.01	0.03 ± 0.01	0.02 ± 0.00	0.02 ± 0.01	0.03 ± 0.01	0.05 ± 0.02	0.02 ± 0.01	0.03 ± 0.01	0.01 ± 0.01	0.02 ± 0.01	0.02 ± 0.01	0.04 ± 0.01
Neutrophils (104·μL^−1^)	0.02 ± 0.01	0.06 ± 0.02	0.06 ± 0.01	0.06 ± 0.01	0.16 ± 0.05	0.18 ± 0.04	0.14 ± 0.04	0.18 ± 0.06	0.09 ± 0.02	0.21 ± 0.07	0.20 ± 0.06	0.25 ± 0.05
2-Way ANOVA												
	Time				Diet				Diet × Time			
	0 h	24 h	48 h		CTR	D1	D2	D3				
Thrombocytes	-	-	-		-	-	-	-	ns			
Lymphocytes	A	B	A		-	-	-	-	ns			
Monocytes	-	-	-		-	-	-	-	ns			
Neutrophils	B	A	A		-	-	-	-	ns			

Different capital letters represent significant differences in time regardless of diet (*p* < 0.05), ns (not significant).

**Table 5:** Relative gene expression profiling of anterior intestine in gilthead seabream juveniles after 1 and 2 weeks of feeding (experiment one). Data are the mean ± SEM (*n* = 12). All data values for each gene were in reference to the expression level of CTR.

		Relative mRNA Expression										
Trial 1	Diets	*il1-β*	*il-34*	*tlr1*	*cd8α*	*igm*	*hepc*	*hsp70*	*gpx*	*sod(mn)*	*muc2*	*muc13*
*1 week*	CTR	1.09 ± 0.15	1.20 ± 0.16	1.05 ± 0.10	1.52 ± 0.43	1.09 ± 0.13	1.58 ± 0.45	1.04 ± 0.09	1.11 ± 0.13	1.06 ± 0.12	1.34 ± 0.29	1.11 ± 0.17
	D1	1.04 ± 0.22	1.18 ± 0.16	1.27 ± 0.12	0.94 ± 0.27	1.17 ± 0.43	1.58 ± 0.52	1.38 ± 0.25	1.49 ± 0.29	1.28 ± 0.14	1.04 ± 0.27	1.27 ± 0.15
	D2	1.41 ± 0.18	1.37 ± 0.12	1.12 ± 0.16	1.22 ± 0.34	2.01 ± 0.63	1.13 ± 0.32	1.67 ± 0.28	1.79 ± 0.26	1.12 ± 0.14	1.35 ± 0.22	1.17 ± 0.14
	D3	1.15 ± 0.09	1.18 ± 0.12	1.28 ± 0.16	1.67 ± 0.58	1.31 ± 0.57	0.99 ± 0.19	1.77 ± 0.38	1.50 ± 0.23	1.46 ± 0.20	1.28 ± 0.18	1.34 ± 0.20
*2 weeks*	CTR	1.54 ± 0.47	1.08 ± 0.15	1.31 ± 0.34	1.23 ± 0.29	1.67 ± 0.50	3.17 ± 1.50	1.04 ± 0.16	1.67 ± 0.41	1.18 ± 0.20	1.93 ± 0.99	1.58 ± 0.58
	D1	1.61 ± 0.62	1.93 ± 0.37	1.94 ± 0.37	1.84 ± 0.42	7.39 ± 3.31	3.45 ± 1.04	1.40 ± 0.25	2.26 ± 0.33	2.25 ± 0.51	3.11 ± 0.68	1.89 ± 0.24
	D2	1.59 ± 0.47	1.43 ± 0.37	2.44 ± 0.65	2.02 ± 0.65	4.16 ± 1.53	3.43 ± 2.61	0.77 ± 0.27	1.78 ± 0.70	1.02 ± 0.29	1.86 ± 0.60	1.59 ± 0.57
	D3	1.31 ± 0.42	1.77 ± 0.54	2.09 ± 0.44	1.70 ± 0.55	3.70 ± 1.58	1.49 ± 0.52	1.56 ± 0.77	1.92 ± 0.50	1.66 ± 0.65	2.19 ± 0.63	1.92 ± 0.69
2 way-ANOVA		***il1-β***	***il-34***	***tlr1***	***cd8α***	***igm***	***hepc***	***hsp70***	***gpx***	***sod(mn)***	***muc2***	***muc13***
*Sig.*	*Time*	ns	ns	ns	0.024	ns	ns	0.009	ns	ns	0.046	ns
	*Diet*	ns	ns	ns	0.747	ns	ns	0.541	ns	ns	0.569	ns
	*Time × Diet*	ns	ns	ns	0.405	ns	ns	0.106	ns	ns	0.156	ns
*Diet*	CTR	-	-	-	-	-	-	-	-	-	-	-
	D1	-	-	-	-	-	-	-	-	-	-	-
	D2	-	-	-	-	-	-	-	-	-	-	-
	D3	-	-	-	-	-	-	-	-	-	-	-
*Time*	*1 week*	-	-	-	B	-	-	B	-	-	B	-
	*2 weeks*	-	-	-	A	-	-	A	-	-	A	-

Different capital letters represent significant differences in time regardless of diet (*p* < 0.05).

**Table 6:** Relative gene expression profiling of anterior intestine in gilthead seabream juveniles following an inflammatory insult after 2 weeks of feeding (experiment two). Data are the mean ± SEM (*n* = 9). All data values for each gene were in reference to the expression level of 0 h CTR fish.

		Relative mRNA Expression										
Trial 2	Diets	*il1-β*	*il-34*	*tlr1*	*cd8α*	*igm*	*hepc*	*hsp70*	*gpx*	*sod(mn)*	*muc2*	*muc13*
*0 h*	CTR	1.54 ± 0.47	1.08 ± 0.15	1.23 ± 0.34	1.31 ± 0.29	1.67 ± 0.50	3.17 ± 1.50	1.04 ± 0.16	1.67 ± 0.41	1.18 ± 0.20	1.93 ± 0.99	1.58 ± 0.58
	D1	1.61 ± 0.62	1.93 ± 0.37	1.84 ± 0.37	1.94 ± 0.42	7.39 ± 3.31	3.45 ± 1.04	1.40 ± 0.25	2.26 ± 0.33	2.25 ± 0.51	3.11 ± 0.68	1.89 ± 0.24
	D2	1.59 ± 0.47	1.43 ± 0.37	2.02 ± 0.65	2.44 ± 0.65	4.16 ± 1.53	3.43 ± 2.61	0.77 ± 0.27 *^#^	1.78 ± 0.70	1.02 ± 0.29	1.86 ± 0.60	1.59 ± 0.57
	D3	1.31 ± 0.42	1.77 ± 0.54	1.70 ± 0.44	2.09 ± 0.55	3.70 ± 1.58	1.49 ± 0.52	1.56 ± 0.77	1.92 ± 0.50	1.66 ± 0.65	2.19 ± 0.63	1.92 ± 0.69
*24 h*	CTR	0.98 ± 0.17	2.20 ± 0.36	2.19 ± 0.38	1.95 ± 0.50	6.18 ± 2.70	2.31 ± 0.83	1.41 ± 0.19 ^a^	1.59 ± 0.61	1.22 ± 0.30	2.98 ± 0.52	1.71 ± 0.31
	D1	0.58 ± 0.22	0.85 ± 0.36	3.47 ± 1.06	0.80 ± 0.37	1.53 ± 0.87	1.77 ± 0.57	0.68 ± 0.22 ^ab^	1.14 ± 0.36	1.27 ± 0.51	3.31 ± 1.19	1.13 ± 0.38
	D2	0.80 ± 0.26	1.75 ± 1.11	2.38 ± 0.59	1.31 ± 0.80	0.77 ± 0.24	1.25 ± 0.41	0.34 ± 0.14 ^b^*	0.89 ± 0.32	0.62 ± 0.25	3.70 ± 2.22	0.94 ± 0.25
	D3	0.96 ± 0.41	1.80 ± 0.57	2.13 ± 0.29	1.95 ± 0.53	4.90 ± 4.01	1.77 ± 0.42	1.25 ± 0.22 ^ab^	0.69 ± 0.20	0.98 ± 0.24	3.06 ± 0.77	2.83 ± 1.22
*48 h*	CTR	0.60 ± 0.07	1.49 ± 0.26	0.90 ± 0.14	2.11 ± 0.55	5.64 ± 1.59	3.70 ± 1.84	3.28 ± 1.47	1.86 ± 0.42	2.40 ± 0.51	2.27 ± 0.52	2.56 ± 0.87
	D1	0.51 ± 0.11	1.08 ± 0.28	1.42 ± 0.33	1.37 ± 0.29	1.11 ± 0.47	1.13 ± 0.34	0.83 ± 0.16	0.84 ± 0.20	0.81 ± 0.22	1.42 ± 0.42	1.02 ± 0.24
	D2	0.84 ± 0.21	1.35 ± 0.20	0.78 ± 0.10	2.51 ± 0.27	1.54 ± 0.34	3.20 ± 0.82	1.12 ± 0.2 ^#^	1.07 ± 0.19	1.27 ± 0.18	2.60 ± 0.47	2.34 ± 0.48
	D3	1.94 ± 1.49	1.44 ± 0.38	1.00 ± 0.27	2.39 ± 1.14	2.59 ± 1.08	4.63 ± 2.31	1.50 ± 0.50	1.64 ± 0.20	1.46 ± 0.60	2.78 ± 0.78	1.75 ± 0.52
Two way-ANOVA		*il1-β*	*il-34*	*tlr1*	*cd8α*	*igm*	*hepc*	*hsp70*	*gpx*	*sod(mn)*	*muc2*	*muc13*
*Sig.*	*Time*	ns	ns	<0.001	ns	ns	ns	0.030	0.006	ns	ns	ns
	*Diet*	ns	ns	ns	ns	ns	ns	<0.001	ns	ns	ns	ns
	*Time × Diet*	ns	ns	ns	ns	ns	ns	0.028	ns	ns	ns	ns
*Diet*	CTR	-	-	-	-	-	-	-	-	-	-	-
	D1	-	-	-	-	-	-	-	-	-	-	-
	D2	-	-	-	-	-	-	-	-	-	-	-
	D3	-	-	-	-	-	-	-	-	-	-	-
*Time*	*0 h*	-	-	B	-	-	-	-	A	-	-	-
	*24 h*	-	-	A	-	-	-	-	B	-	-	-
	*48 h*	-	-	B	-	-	-	-	AB	-	-	-

Different superscript letters represent significant differences between diets within the same time (*p* < 0.05). Different superscript symbols represent significant differences in time within the same diet (*p* < 0.05). Different capital letters represent significant differences in time regardless of diet (*p* < 0.05).

**Table 7:** Microalgae *Chlorella vulgaris* biomass composition (prior to extraction).

Nutrients	Quantity (g/100 g)
Crude Protein	52.2
Crude Fat	7.9
Carbohydrates	10.9
Fibers	15.5
Mineral matter	11.1
Moisture	2.4

**Table 8:** *Chlorella vulgaris* soluble extract protein concentration and in vitro bioactivities.

	*Chlorella vulgaris* Soluble Extract
% Protein	44.71 ± 1.75
Antioxidant activity (ORAC) (µmol TE/g of extract)	462.83 ± 39.97
Anti-hypertensive activity (iACE) (IC_50_ µg protein mL^−1^)	286.0 ± 55.00
Anti-diabetic activity (% of inhibition of α-Glucosidase enzyme in a solution with 30 mg mL^−1^ of soluble extract)	31.36 ± 3.90

**Table 9:** Ingredients and proximate composition of experimental diets.

Ingredients (%)	CTR	D1	D2	D3
Fishmeal Super Prime ^1^	10.00	10.00	10.00	10.00
Fish gelatin ^2^	2.00	2.00	2.00	2.00
Soy protein concentrate ^3^	10.00	10.00	10.00	10.00
Wheat gluten ^4^	7.00	7.00	7.00	7.00
Corn gluten ^5^	15.00	15.00	15.00	15.00
Soybean meal ^6^	20.00	20.00	20.00	20.00
Rapeseed meal ^7^	5.25	5.25	5.25	5.25
Sunflower meal ^8^	5.00	5.00	5.00	5.00
Wheat meal ^9^	7.00	5.00	7.00	7.00
Fish oil ^10^	4.90	4.90	4.90	4.90
Soybean oil ^11^	9.10	9.10	9.10	9.10
Premix 1% ^12^	1.00	1.00	1.00	1.00
Binder (Celatom—Diatomite) ^13^	1.00	1.00	1.00	1.00
MAP (Monoammonium phosphate) ^14^	1.50	1.50	1.50	1.50
L-Lysine ^15^	1.00	1.00	1.00	1.00
L-Threonine ^16^	0.10	0.10	0.10	0.10
DL-Methionine ^17^	0.15	0.15	0.15	0.15
Chlorella whole biomass—Algafarm ^18^	0.00	2.00	0.00	0.00
Chlorella—soluble fraction ^19^	0.00	0.00	0.10	0.00
Chlorella—Insoluble residue ^20^	0.00	0.00	0.00	0.10

^1^ 66.3% CP, 11.5% CF, Pesquera Diamante, Peru; ^2^ 94% WEISHARDT, Slovakia; ^3^ 62.2% CP, 0.7% CF, Soycomil P, ADM, Netherlands; ^4^ 80.4% CP, 5.8% CF, VITAL, Roquette, France; ^5^ 61.2% CP, 5.2% CF, COPAM, Portugal; ^6^ Dehulled solvent extracted: 47.4% CP, 2.6% CF, Cargill, Spain; ^7^ Solvent extracted: 34.3% CP, 2.1% CF, Ribeiro e Sousa Lda, Portugal; ^8^ Solvent extracted: 29.1% CP, 1.8% CF, Ribeiro e Sousa Lda, Portugal; ^9^ 11.7% CP, 1.6% CF, Molisur, Spain; ^10^ 98.1% CF (16% EPA; 12% DHA), Sopropêche, France; ^11^ 98.6%, JC Coimbra, Portugal; ^12^ Vitamins (IU or mg/Kg diet): DL-alphatocopherol acetate, 100 mg; sodium menadione bisulphate, 25 mg; retinyl acetate, 20,000 IU; DL-cholecalciferol, 2000 IU; thiamine, 30 mg; riboflavin, 30 mg; pyridoxine, 20 mg; cyanocobalamin, 0.1 mg; nicotidin acid, 200 mg; folic acid, 15 mg; ascorbic acid, 1000 mg; inositol, 500 mg; biotin, 3 mg; calcium panthotenate, 100 mg; choline chloride, 1000 mg, betaine, 500 mg. Minerals (g or mg/kg diet): cobalt carbonate, 0.65 mg; copper sulphate, 9 mg; ferric sulphate, 6 mg; potassium iodide, 0.5 mg; manganese oxide, 9.6 mg; sodium selenite, 0.01 mg; zinc sulphate. 7.5 mg; sodium chloride, 400 mg; calcium carbonate, 1.86 g; excipient wheat middling’s, Premix Lda, Portugal; ^13^ CELATOM FP1SL (diatomite), Angelo Coimbra S.A., Portugal; ^14^ Windmill AQUAPHOS (26% P), ALIPHOS, Netherlands; ^15^ 99% Lys, Ajinomoto EUROLYSINE S.A.S, France; ^16^ 98.5% Thr, Ajinomoto EUROLYSINE S.A.S, France; ^17^ 99% Met, Rodhimet NP99, ADISSEO, France; ^18^ *Chlorella vulgaris* lyophilized biomass, Allmicroalgae, Portugal; ^19,20^ *Chlorella vulgaris* aqueous and insoluble extracts, CBQF—Escola Superior de Biotecnologia, Universidade Católica Portuguesa, Portugal.

**Table 10:** PCR-array layout for gene expression profiling of anterior gut in sea bream.

Function	Gene	Symbol	Accession Number
Intestinal epithelium protection	Mucin 2	*muc2*	JQ277710
	Mucin 13	*muc13*	JQ277713
Cytokines	Interleukin 1 beta	*il1b*	AJ277166.2
	Interleukin 34	*Il34*	JX976629.1
Pattern recognition receptors	Toll like receptor 1	*tlr1*	KF857322
Cell markers	CD8 alpha	*cd8α*	AJ878605
Antibodies	Immunoglobulin M	*igm*	AM493677
Antimicrobial defence/Iron recycling	Hepcidin	*hepc*	EF625901
Oxidative stress defences	Heat-shock protein 70	*hsp70*	DQ524995.1
	Glutathione peroxidase	*gpx*	DQ524992
	Manganese superoxide dismutase	*Sod(mn)*	JQ308833
Reference genes	Elongation factor 1α	*ef1α*	AF184170
	Ribosomal protein 18S	*rps18*	AM490061

## Data Availability

Not applicable.

## Supplementary Materials

The following supporting information can be downloaded at: https://www.mdpi.com/article/10.3390/md20070407/s1, Table S1. Relative gene expression profiling of anterior intestine in gilthead seabream juveniles fed experimental diets.; Figure S1. Protein/peptide profile of *C. vulgaris* hydrolysate, showing the main molecular weight ranges, the area of the main peak, and the localization of all the 42 identified peaks.
