# Supplementary material for: Chlorella vulgaris Extracts as Modulators of the Health Status and the Inflammatory Response of Gilthead Seabream Juveniles (Sparus aurata)
Source: Mar Drugs. 2022 Jun 21;20(7):407. doi: 10.3390/md20070407 (PMC9323325; doi:10.3390/md20070407)
Supplement: Supplementary file 1 [file marinedrugs-20-00407-s001.zip › marinedrugs-1726009-supplementary.pdf]

Supplementary Materials

Table S1. Primers for qPCR amplification in seabream gut.

Gene	Symbol	Annealing T °C	Primer sequence
Mucin 2	*muc2*	60	F: ACGCTTCAGCAATCGCACCAT<br>R: CCACAACCACACTCCTCCACAT
Mucin 13	*muc13*	60	F: TTCAAACCCGTGTGGTCCAG<br>R: GCACAAGCAGACATAGTTCGGATAT
Interleukin-1 beta	*il1β*	60	F: TCTTCAAATTCCTGCCACCA<br>R: CAATGCCACCTTGTGGTGAT
Interleukin-34	*Il34*	60	F: CATCAGGGTTCATCACAACG<br>R: GACTCCCTCTGCATCCTTGA
Toll like receptor 1	*tlr1*	60	F: GGGACCTGCCAGTGTGTAAC<br>R: GCGTGGATAGAGTTGGACTTGAG
CD8 alpha	*cd8α*	60	F: CTCGACTGGTCGGAGTTAA<br>R: TCCATCAGCGGCTGCTCGT
Immunoglobulin M	*igm*	59	F: CAGCCTCGAGAAGTGGAAAC<br>R: GAGGTTGACCAGGTTGGTGT
Hepcidin	*hepc*	60	F: GCCATCGTGCTCACCTTTAT<br>R: CCTGCTGCCATACCCCATCTT
Heat-shock protein 70	*hsp70*	55	F: ACGGCATCTTTGAGGTGAAG<br>R: TGGCTGATGTCCTTCTTGTG
Glutathione peroxidase	*gpx*	60	F: GAAGGTGGATGTGAATGGAAAAGATG<br>R: CTGACGGGACTCCAAATGATGG
Manganese superoxide dis-mutase	*Sod(mn)*	60	F: CCTGACCTGACCTACGACTATGG<br>R: AGTGCCTCCTGATATTTCTCCTCTG
Elongation factor 1α	*ef1α*	58	F: CTGTCAAGGAAATCCGTCGT<br>R: TGACCTGAGCGTTGAAGTTG
Ribosomal protein 18S	*rps18*	60	F: AGGGTGTTGGCAGACGTTAC<br>R: CTTCTGCCTGTTGAGGAACC

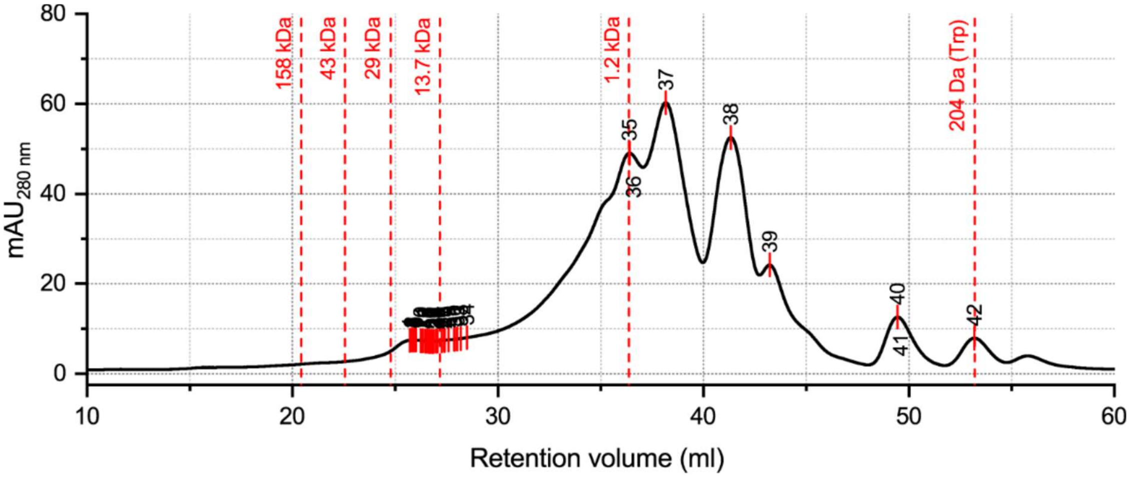

Figure S1. Protein/peptide profile of *C. vulgaris* peptide-enriched extract. Main molecular weight ranges, area of the main peak and the localization of all identified peaks as previously reported in Cunha, et al. [41].
